# Reflections on the 1976 Swine Flu Vaccination Program

**DOI:** 10.3201/eid1201.051007

**Published:** 2006-01

**Authors:** David J. Sencer, J. Donald Millar

**Affiliations:** * Atlanta, Georgia, USA;; † Murraysville, Georgia, USA

**Keywords:** influenza, swine flu, Guillain Barré syndrome, vaccination, decision-making, history

## Abstract

In 1976, 2 recruits at Fort Dix, New Jersey, had an influenzalike illness. Isolates of virus taken from them included A/New Jersey/76 (Hsw1n1), a strain similar to the virus believed at the time to be the cause of the 1918 pandemic, commonly known as swine flu. Serologic studies at Fort Dix suggested that >200 soldiers had been infected and that person-to-person transmission had occurred. We review the process by which these events led to the public health decision to mass-vaccinate the American public against the virus and the subsequent events that led to the program's cancellation. Observations of policy and implementation success and failures are presented that could help guide decisions regarding avian influenza.

## Abstract 2

The swine flu vaccination program has implications for the current pandemic preparedness.

"Flu to the Starboard! Man the Harpoons! Fill with Vaccine! Get the Captain! Hurry!"

**Edwin D. Kilbourne, New York Times, February 13, 1976** (*1*)

"Grounding a Pandemic"

**Barack Obama and Richard Lugar, New York Times, June 6, 2005** (*2*)

"It has been 37 years since the last influenza pandemic, or widespread global epidemic, so by historic patterns we may be due for another."

**New York Times, July 17, 2005** (*3*)

Kilbourne in 1976 (*1*) noted that pandemics of influenza occur every 11 years. Since the latest prediction in the New York Times (*3*) suggests that after 39 years we may be overdue for a pandemic, and since 2 US senators have recently headlined the possibility (*2*), that observation may become a political fact. Whether it becomes a scientific fact and a policy fact is yet to be seen. Some reflections on 1976 from 2 insiders' viewpoints may identify some of the pitfalls that public health policymakers will face in addressing potential influenza pandemics.

## Swine Flu at Fort Dix

On February 3, 1976, the New Jersey State Health Department sent the Center for Disease Control (CDC) in Atlanta isolates of virus from recruits at Fort Dix, New Jersey, who had influenzalike illnesses. Most of the isolates were identified as A/Victoria/75 (H3N2), the contemporary epidemic strain. Two of the isolates, however, were not typeable in that laboratory. On February 10, additional isolates were sent and identified in CDC laboratories as A/New Jersey/76 (Hsw1N1), similar to the virus of the 1918 pandemic and better known as "swine flu."

A meeting of representatives of the military, the National Institute of Health, the Food and Drug Administration (FDA), and the State of New Jersey Department of Health was quickly convened on Saturday, February 14, 1976. Plans of action included heightened surveillance in and around Fort Dix, investigation of the ill recruits to determine if contact with pigs had occurred, and serologic testing of recruits to determine if spread had occurred at Fort Dix.

Surveillance activities at Fort Dix gave no indication that recruits had contact with pigs. Surveillance in the surrounding communities found influenza caused by the current strain of influenza, A/Victoria, but no additional cases of swine flu. Serologic testing at Fort Dix indicated that person-to-person transmission had occurred in >200 recruits (*4*).

In 1974 and 1975, 2 instances of humans infected with swine influenza viruses had been documented in the United States. Both persons involved had close contact with pigs, and no evidence for spread of the virus beyond family members with pig contact could be found (*5*).

## The National Influenza Immunization Program

On March 10, 1976, the Advisory Committee on Immunization Practices of the United States Public Health Service (ACIP) reviewed the findings. The committee concluded that with a new strain (the H1N1 New Jersey strain) that could be transmitted from person to person, a pandemic was a possibility. Specifically, the following facts were of concern: 1) persons <50 years of age had no antibodies to this new strain; 2) a current interpandemic strain (A/Victoria) of influenza was widely circulating; 3) this early detection of an outbreak caused by A/New Jersey/76/Hsw1N1 (H1N1) provided an opportunity to produce a vaccine since there was sufficient time between the initial isolates and the advent of an expected influenza season to produce vaccine. In the past when a new pandemic strain had been identified, there had not been enough time to manufacture vaccine on any large scale; 4) influenza vaccines had been used for years with demonstrated safety and efficacy when the currently circulating vaccine strain was incorporated; 5) the military vaccine formulation for years had included H1N1, an indication that production was possible, and no documented adverse effects had been described.

ACIP recommended that an immunization program be launched to prevent the effects of a possible pandemic. One ACIP member summarized the consensus by stating "If we believe in prevention, we have no alternative but to offer and urge the immunization of the population." One ACIP member expressed the view that the vaccine should be stockpiled, not given.

Making this decision carried an unusual urgency. The pharmaceutical industry had just finished manufacture of the vaccine to be used in the 1976–1977 influenza season. At that time, influenza vaccine was produced in fertilized hen's eggs from special flocks of hens. Roosters used for fertilizing the hens were still available; if they were slaughtered, as was customary, the industry could not resume production for several months.

On March 13, an action memo was presented to the Secretary of the Department of Health Education and Welfare (DHEW). It outlined the problem and presented 4 alternative courses of action. First was "business as usual," with the marketplace prevailing and the assumption that a pandemic might not occur. The second was a recommendation that the federal government embark on a major program to immunize a highly susceptible population. As a reason to adopt this plan of action, the memo stated that "the Administration can tolerate unnecessary health expenditures better than unnecessary death and illness if a pandemic should occur." The third proposed course of action was a minimal response, in which the federal government would contract for sufficient vaccine to provide for traditional federal beneficiaries—military personnel, Native Americans, and Medicare-eligible persons. The fourth alternative was a program that would represent an exclusively federal response without involvement of the states.

The proposal recommended by the director of CDC was the second course, namely, for the federal government to contract with private pharmaceutical companies to produce sufficient vaccine to permit the entire population to be immunized against H1N1. The federal government would make grants to state health departments to organize and conduct immunization programs. The federal government would provide vaccine to state health departments and private medical practices. Since influenza caused by A/Victoria was active worldwide, industry was asked to incorporate the swine flu into an A/Victoria product to be used for populations at high risk.

Before the discussions with the secretary of DHEW had been completed, a member of his staff sent a memo to a health policy advisor in the White House, raising the specter of the 1918 pandemic, which had been specifically underemphasized in the CDC presentation. CDC's presentation highlighted the pandemic potential, comparing it with the 1968–69 Hong Kong and 1957–58 Asian pandemics. President Gerald Ford's staff recommended that the president convene a large group of well-known and respected scientists (Albert Sabin and Jonas Salk had to be included) and public representatives to hear the government's proposal and make recommendations to the president about it. After the meeting, the president had a press conference, highlighted by the unique simultaneous appearance of Salk and Sabin. President Ford announced that he accepted the recommendations that CDC had originally made to the secretary of DHEW. The National Influenza Immunization Program (NIIP) was initiated.

The proposal was presented to 4 committees of the Congress, House and Senate authorization committees and House and Senate appropriation committees. All 4 committees reported out favorable legislation, and an appropriation bill was passed and signed.

The estimated budgeted cost of the program was $137 million. When Congress passed the appropriation, newspapers mischaracterized the cost as "$1.9 billion" because the $137 million was included as part of a $1.9 billion supplemental appropriation for the Department of Labor. In the minds of the public, this misconception prevailed.

Immediately after the congressional hearing, a meeting of all directors of state health departments and medical societies was held at CDC. The program was presented by CDC, and attendees were asked for comments. A representative from the New Jersey state health department opposed the plan; the Wisconsin state medical society opposed any federal involvement. Otherwise, state and local health departments approved the plan.

Within CDC, a unit charged with implementing the program, which reported to the director, was established. This unit, NIIP, had complete authority to draw upon any resources at CDC needed. NIIP was responsible for relations with state and local health departments (including administration of the grant program for state operations, technical advice to the procurement staff for vaccine, and warehousing and distribution of the vaccine to state health departments) and established a proactive system of surveillance for possible adverse effects of the influenza vaccines, the NIIP Surveillance Assessment Center (NIIP-SAC). (This innovative surveillance system would prove to be NIIP's Trojan horse.) In spite of the obstacles discussed below, NIIP administered a program that immunized 45 million in 10 weeks, which resulted in doubling the level of immunization for persons deemed to be at high risk, rapidly identifying adverse effects, and developing and administering an informed consent form for use in a community-based program.

## Obstacles to the Vaccination Plan

The principal obstacle was the lack of vaccines. As test batches were prepared, the largest ever field trials of influenza vaccines ensued. The vaccines appeared efficacious and safe (although in the initial trials, children did not respond immunologically to a single dose of vaccine, and a second trial with a revised schedule was needed) (*6*). Hopes were heightened for a late summer/early fall kickoff of mass immunization operations.

In January 1976, before the New Jersey outbreak, CDC had proposed legislation that would have compensated persons damaged as a result of immunization when it was licensed by FDA and administered in the manner recommended by ACIP. The rationale given was that immunization protects the community as well as the individual (a societal benefit) and that when a person participating in that societal benefit is damaged, society had a responsibility to that person. The proposal was sent back from a staff member in the Surgeon General's office with a handwritten note, "This is not a problem."

Soon, however, NIIP received the first of 2 crippling blows to hopes to immunize "every man, woman, and child." The first was later in 1976, when instead of boxes of bottled vaccine, the vaccine manufacturers delivered an ultimatum—that the federal government indemnify them against claims of adverse reactions as a requirement for release of the vaccines. The government quickly capitulated to industry's demand for indemnification. While the manufacturers' ultimatum reflected the trend of increased litigiousness in American society, its unintended, unmistakable subliminal message blared "There's something wrong with this vaccine." This public misperception, warranted or not, ensured that every coincidental health event that occurred in the wake of the swine flu shot would be scrutinized and attributed to the vaccine.

On August 2, 1976, deaths apparently due to an influenzalike illness were reported from Pennsylvania in older men who had attended the convention of the American Legion in Philadelphia. A combined team of CDC and state and local health workers immediately investigated. By the next day, epidemiologic evidence indicated that the disease was not influenza (no secondary cases occurred in the households of the patients). By August 4, laboratory evidence conclusively ruled out influenza. However, this series of events was interpreted by the media and others as an attempt by the government to "stimulate" NIIP.

Shortly after the national campaign began, 3 elderly persons died after receiving the vaccine in the same clinic. Although investigations found no evidence that the vaccine and deaths were causally related, press frenzy was so intense it drew a televised rebuke from Walter Cronkite for sensationalizing coincidental happenings.

### Guillain-Barré Syndrome

What NIIP did not and could not survive, however, was the second blow, finding cases of Guillain-Barré syndrome (GBS) among persons receiving swine flu immunizations. As of 1976, >50 "antecedent events" had been identified in temporal relationship to GBS, events that were considered as possible factors in its cause. The list included viral infections, injections, and "being struck by lightning." Whether or not any of the antecedents had a causal relationship to GBS was, and remains, unclear. When cases of GBS were identified among recipients of the swine flu vaccines, they were, of course, well covered by the press. Because GBS cases are always present in the population, the necessary public health questions concerning the cases among vaccine recipients were "Is the number of cases of GBS among vaccine recipients higher than would be expected? And if so, are the increased cases the result of increased surveillance or a true increase?" Leading epidemiologists debated these points, but the consensus, based on the intensified surveillance for GBS (and other conditions) in recipients of the vaccines, was that the number of cases of GBS appeared to be an excess.

Had H1N1 influenza been transmitted at that time, the small apparent risk of GBS from immunization would have been eclipsed by the obvious immediate benefit of vaccine-induced protection against swine flu. However, in December 1976, with >40 million persons immunized and no evidence of H1N1 transmission, federal health officials decided that the possibility of an association of GBS with the vaccine, however small, necessitated stopping immunization, at least until the issue could be explored. A moratorium on the use of the influenza vaccines was announced on December 16; it effectively ended NIIP of 1976. Four days later the New York Times published an op-ed article that began by asserting, "Misunderstandings and misconceptions... have marked Government ... during the last eight years," attributing NIIP and its consequences to "political expediency" and "the self interest of government health bureaucracy" (*7*). These simple and sinister innuendos had traction, as did 2 epithets used in the article to describe the program, "debacle" in the text and "Swine Flu Fiasco" in the title.

On February 7, the new secretary of DHEW, Joseph A. Califano, announced the resumption of immunization of high-risk populations with monovalent A/Victoria vaccine that had been prepared as part of the federal contracts, and he dismissed the director of CDC.

## Lessons Learned

NIIP may offer lessons for today's policymakers, who are faced with a potential pandemic of avian influenza and struggling with decisions about preventing it (Table). Two of these lessons bear further scrutiny here.

**Table:** Lessons learned from the 1976 National Influenza Immunization Program (NIIP).

1. Expect the unexpected: it will always happen. Some examples:
• Children did not respond to the initial formulation of vaccine. • Liability for untoward events after immunization became a major issue. • Deaths occurred in Pittsburgh that were coincidental with but unrelated to the vaccines (*8*). • Cases of a new and unrelated disease, Legionnaires disease, appeared (*9*). • "Excess" cases of Guillain-Barré syndrome appeared among recipients of vaccines (*10*). • Erroneous laboratory reports of viral isolates or serologic conversions occurred in Washington, DC, Boston, Virginia, and Taiwan. • The pandemic failed to appear.
2. Surveillance for influenza disease worked well. This was plain, "old-fashioned" surveillance without computers. A new strain of influenza was identified within weeks of the first recognized outbreak of illness.
3. Interagency cooperation works without formal agreements. The state health departments, military, National Institutes of Health, US Food and Drug Administration, and Center for Disease Control all worked together in a cooperative and mutually beneficial manner.
4. Surveillance for untoward events demonstrated that only when large numbers of people are exposed to a vaccine or drug are adverse reactions identified (Guillain-Barré syndrome with influenza vaccines; paralysis with the Cutter poliovirus vaccine in 1955).
5. Health legislation can and should be developed on the basis of the epidemiologic picture.
6. Media and public awareness can be a major obstacle to implementing a large, innovative program responding to risks that are difficult, if not impossible, to quantitate.
• Creating a program as a presidential initiative makes modifying or stopping the program more difficult. • Explanations should be communicated by those who can give authoritative scientific information. • Periodic press briefings work better than responding to press queries.
7. The advisability of the decision to begin immunization on the strength of the Fort Dix episode is worthy of serious question and debate (see text).
8. The risk of potentially unnecessary costs in a mass vaccination campaign is minimal. (The direct cost of the 1976 program was $137 million. In today's dollars, this is <$500 million.) The potential cost of a pandemic is inestimable but astronomical.

### Media and Presidential Attention

While all decisions related to NIIP had been reached in public sessions (publishing of the initial virus findings in CDC's weekly newsletter, the Morbidity and Mortality Weekly Report (MMWR); New York Times reporter Harold Schmeck's coverage of the ACIP sessions, the president's press conference, and 4 congressional hearings), effective communication from scientifically qualified persons was lacking, and the perception prevailed that the program was motivated by politics rather than science. In retrospect (and to some observers at the time), the president's highly visible convened meeting and subsequent press conference, which included pictures of his being immunized, were mistakes. These instances seemed to underline the suspicion that the program was politically motivated, rather than a public health response to a possible catastrophe.

Annex 11 of the draft DHEW pandemic preparedness plan states, "For policy decisions and in communication, making clear what is not known is as important as stating what is known. When assumptions are made, the basis for the assumptions and the uncertainties surrounding them should be communicated" (*11*). This goal is much better accomplished if the explanations are communicated by those closest to the problem, who can give authoritative scientific information. Scientific information coming from a nonscientific political figure is likely to encourage skepticism, not enthusiasm.

Neither CDC nor the health agencies of the federal government had been in the habit of holding regular press conferences. CDC considered that its appropriate main line of communication was to states and local health departments, believing that they were best placed to communicate with the public. MMWR served both a professional and public audience and accounted for much of CDC's press coverage. In 1976, no all-news stations existed, only the nightly news. The decision to stop the NIIP on December 16, 1976, was announced by a press release from the office of the assistant secretary for health. The decision to reinstitute the immunization of those at high risk was announced by a press release from the office of the secretary, DHEW. In retrospect, periodic press briefings would have served better than responding to press queries. The public must understand that decisions are based on public health, not politics. To this end, health communication should be by health personnel through a regular schedule of media briefings.

### Decision To Begin Immunization

This decision is worthy of serious question and debate. As Walter Dowdle (*12*) points out in this issue of Emerging Infectious Diseases, the prevailing wisdom was that a pandemic could be expected at any time. Public health officials were concerned that if immunization was delayed until H1N1 was documented to have spread to other groups, the disease would spread faster than any ability to mobilize preventive vaccination efforts. Three cases of swine influenza had recently occurred in persons who had contact with pigs. In 1918, after the initial outbreak of influenza at Fort Riley in April, widespread outbreaks of influenza did not occur until late summer (*13*).

The Delphi exercise of Schoenbaum in early fall of 1976 (*13*) was the most serious scientific undertaking to poll scientists to decide whether or not to continue the program. Its main finding was that the cost benefit would be best if immunization were limited to those >25 years of age (and now young children are believed to be a potent source of spread of influenza virus!). Unfortunately, no biblical Joseph was there to rise from prison and interpret the future.

As Dowdle further states (*12*), risk assessment and risk management are separate functions. But they must come together with policymakers, who must understand both. These discussions should not take place in large groups in the president's cabinet room but in an environment that can establish an educated understanding of the situation. Once the policy decisions are made, implementation should be left to a single designated agency. Advisory groups should be small but representative. CDC had the lead responsibility for operation of the program. Implementation by committee does not work. Within CDC, a unit was established for program execution, including surveillance, outbreak investigation, vaccine procurement and distribution, assignment of personnel to states, and awarding and monitoring grants to the states. Communications up the chain of command to the policymakers and laterally to other directly involved federal agencies were the responsibility of the CDC director, not the director of NIIP, who was responsible for communications to the states and local health departments, those ultimately implementing operations of the program. This organizational mode functioned well, a tribute to the lack of interagency jealousies.

### Decision-making Risks

When lives are at stake, it is better to err on the side of overreaction than underreaction. Because of the unpredictability of influenza, responsible public health leaders must be willing to take risks on behalf of the public. This requires personal courage and a reasonable level of understanding by the politicians to whom these public health leaders are accountable. All policy decisions entail risks and benefits: risks or benefits to the decision maker; risks or benefits to those affected by the decision. In 1976, the federal government wisely opted to put protection of the public first.
